# Agri-Nanotechnology and Tree Nanobionics: Augmentation in Crop Yield, Biosafety, and Biomass Accumulation

**DOI:** 10.3389/fbioe.2022.853045

**Published:** 2022-04-26

**Authors:** Manzar Abbas, Kuan Yan, Jia Li, Sara Zafar, Zuhair Hasnain, Nazia Aslam, Naeem Iqbal, Syed Sarfaraz Hussain, Muhammad Usman, Mubashir Abbas, Muhammad Tahir, Sammar Abbas, Saqi Kosar Abbas, Huang Qiulan, Xianming Zhao, Ahmed H. El-Sappah

**Affiliations:** ^1^ School of Agriculture, Forestry and Food Engineering, Yibin University, Yibin, China; ^2^ Government College University, Faisalabad, Pakistan; ^3^ PMAS Arid Agriculture University, Rawalpindi, Pakistan; ^4^ State Key Laboratory of Tree Genetics and Breeding, Chinese Academy of Forestry, Beijing, China; ^5^ Beijing Advanced Innovation Center for Tree Breeding by Molecular Design, National Engineering Laboratory for Tree Breeding, College of Biological Sciences and Technology, Beijing Forestry University, Beijing, China; ^6^ National Institute for Biotechnology and Genetic Engineering College, Pakistan Institute of Engineering and Applied Sciences (NIBGE-C, PIEAS), Faisalabad, Pakistan; ^7^ Faculty of Veterinary and Animal Sciences, MNS University of Agriculture, Multan, Pakistan; ^8^ Biotechnology Research Institute, Chinese Academy of Agricultural Sciences, Beijing, China; ^9^ Beijing Advanced Innovation Center for Tree Breeding by Molecular Design, National Engineering Laboratory for Tree Breeding, Key Laboratory of Genetics and Breeding in Forest Trees and Ornamental Plants, Ministry of Education, College of Biological Sciences and Biotechnology, Beijing Forestry University, Beijing, China; ^10^ College of Biological Sciences and Biotechnology, Beijing Forestry University, Beijing, China; ^11^ College of Agriculture, BZU, Bahadur Sub-Campus Layyah, Layyah, Pakistan; ^12^ Department of Genetics, Faculty of Agriculture, Zagazig University, Zagazig, Egypt

**Keywords:** nanosensors, nanofertilizer, nanopesticides, nanobionics, biosafety, biomass accumulation

## Abstract

Nanomaterials (NMs) are the leading edge as an amazing class of materials that consists of at least one dimension in the range of 1–100 nm. NMs can be made with exceptional magnetic, electrical, and catalytic properties different from their bulk counterparts. We summarized unique features of NMs, their synthesis, and advances in agri-nanotechnology and cutting-edge nanobionics. The review describes advances in NMs including their applications, dosimetry to ensure biosafety, remote sensing of agro-forestry fields, nanofertilizers, and nanopesticides, and avoid post-harvest losses, gene delivery, and nanobionics. Tree nanobionics has enabled the synthesis and delivery of nanosensors, which enhance the rate of photosynthesis, detection of pathogens, and poisonous residues to ensure biosafety and biomass accumulation. Finally, we conclude by discussing challenges, future perspectives, and agro-ecological risks of using NMs.

## Graphical Abstract

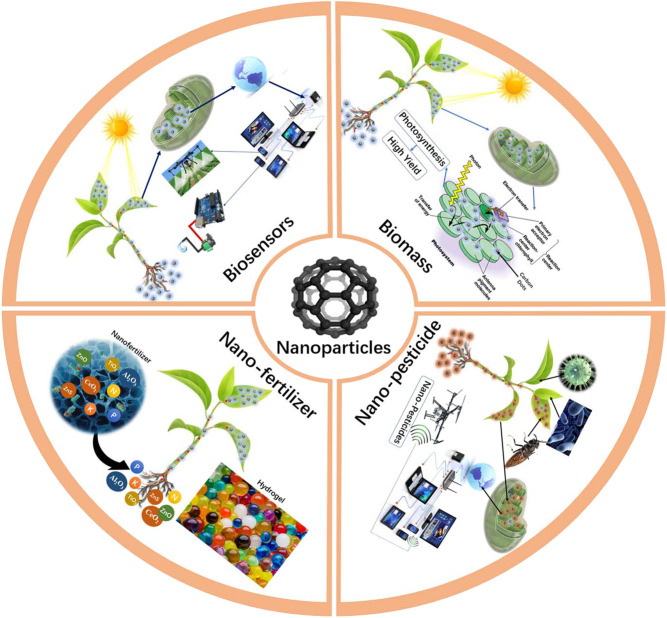

## Introduction

The term “nano” is used to describe one-billionth or 10^–9^. Nanoparticles (NPs) possess three external nanoscale dimensions. Bio-nanotechnology is one of the most advanced technologies dealing with particles in the nanometer (nm) range, which has numerous lucrative properties like small size, large surface area, and high penetration capacity, crossing different cellular barriers, and apart from that, their biocompatible nature makes them more suitable for a number of applications like bioimaging, biosensing, targeted drug delivery, and immunomodulation (Table 1). Living organisms due to their cellular composition are somewhat bigger, of almost 10 µm, and their organelles are in the nanometer range. The smaller proteins of just 5 nm in size are even bigger than the smallest novel NPs. This size comparison urges us to think of nanoparticles’ importance in their working inside the cells without any hindrance.

**TABLE 1:** Size, examples, and applications of 0D, 1D, 2D, and 3D nanomaterials.

Nanomaterial	Examples	Applications	References
0D-NMs: ultra-small size <100 nm, high surface to volume ratio	Quantum dots, magnetic NPs, polymer dots, and noble metal NPs	Fabrication of photocatalysts, doping of semiconductors, solar cells, piezoelectric energy transformation, biosensors, and drug delivery	Nandwana et al. (2018)
			Huang et al. (2019)
			Wang et al. (2020)
1D-NMs: small size <100 nm, one scale dimension	Nanowires, nanorods, and nanobelts	Surface coatings, packaging of IT systems, biological sensors, magneto-optic, nano-electronics, nano-optical devices, Nano-chemical sensors, and nano-fiberoptic systems	Ngô and Van de Voorde, (2014)
			Arrabito et al. (2020)
			Arrabito et al. (2020)
2D-NMs: small size <100 nm, two dimensions in nanometer range	Nano-fibrils, nano-dendrimers, nano-tubes, and nano-fibers	Nanodevices, nanocontainers, nanoreactors, nanotemplates, photocatalysts, doping of semiconductors, electrodes, and ultrathin and smooth films	Tiwari et al. (2012)
			Zhu et al. (2017)
			Sinha et al. (2021)
3D-NMs: size <100 nm in all dimensions, three dimensions in nanometers	Hollow spheres, nano-balls, nano-coils, nano-cones, and nano-pillars	Electrodes of batteries, magnetic materials, catalysis, and transport of molecules	Tiwari et al. (2012)

There are different types of nanostructured materials: 1) carbon-based nanomaterials: composed of carbon and present in the form of carbon black, spheres, nanofibers, or ellipsoids, that is, carbon nanofibers, graphene, fullerenes (C60), carbon nanotubes, and carbon black (Kumar and Kumbhat, 2016; Baig et al., 2021); 2) organic/inorganic-based nanomaterials: made of organic matter called organic-based, while those without carbon are called inorganic-based NMs, and non-covalent (weak) interactions are mostly used for the preparation of organic NMs into required structures like liposomes, polymer, micelles, and dendrimers; 3) composite-based nanomaterials: these are the multiphase NPs and NSMs with one phase on the nanoscale dimension, which is either the combination of NPs with other NPs or NPs with larger or metal–organic frameworks or with bulk-type materials. The composites are formed by the combination of metal-based, organic-based, or carbon-based NMs with any form of ceramic, polymer bulk materials, or metal. Morphologies of the NMs synthesized by the different processes depend upon their applications. Dimensions of the nanomaterials classify under different categories (Table 1) (Jeevanandam et al., 2018).

**FIGURE 1:**
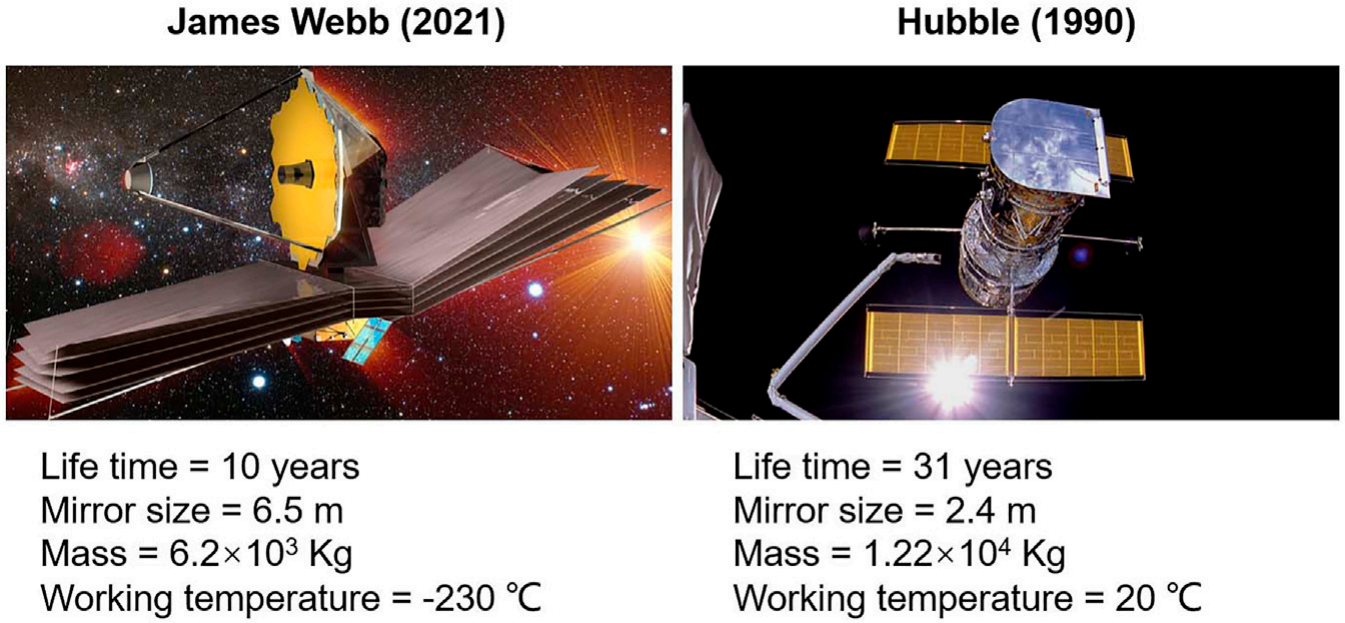
Comparative efficacy of James Webb telescope.

The terms nanoplate or nanorod are in use instead of nanoparticles (NPs) when the nano-objects are with the shortest and longest axis lengths. Two groups of NPs are being synthesized: organic and inorganic. Magnetic NPs belong to inorganic NPs, which can easily be modified in a magnetic field (Natarajan et al., 2019). Materials after conversion to nanoscale exhibit properties like superparamagnetic behavior, chemical reactivity, extraordinary strength, and electrical conductivity (Table 1). Due to unique physiochemical properties and large surface area, gold nanoparticles (Au-NPs) can be transformed into highly efficient nanosensors. Recently, a Au-NP-plated highly efficient James Webb telescope was launched in 2021 harboring a three-time big mirror but half in weight as compared to its predecessor—the Hubble telescope (Figure 1). Au-NPs are less toxic and biologically compatible to be used as nanosensors in plant research (Youssef and El-Sayed, 2018). Similarly, SiO_2_ and Ag-NPs are being used at a large scale for filtration and sterile food packaging (Wang et al., 2021).

Phyto-nanotechnology or agri-nanotechnology deals with the use of smallest particles, especially at the atomic level in plant science research. This is because nanotechnology for agricultural applications will have to address the large-scale inherent imperfections and complexities of farm production systems (extremely low input use efficiency) that might require nanomaterials with flexible dimensions, which nevertheless perform tasks efficiently in agricultural production systems. Agri-nanotechnology is being used for targeted gene delivery, drug delivery, fertilizer delivery, biosensing, and bioimaging purposes.

## Synthesis of Nanoparticles

The leaves of different plants are being used for the biosynthesis of NPs such as leaves of guava, bitter, moringa, and scent. NPs are also being synthesized using processes like alloys of bimetal, metal oxides, and noble metals. Green synthesis is usually preferred due to the easy availability of plants and their non-toxic and eco-friendly nature (Zafar et al., 2021).

### Top-Down Approaches

**FIGURE 2:**
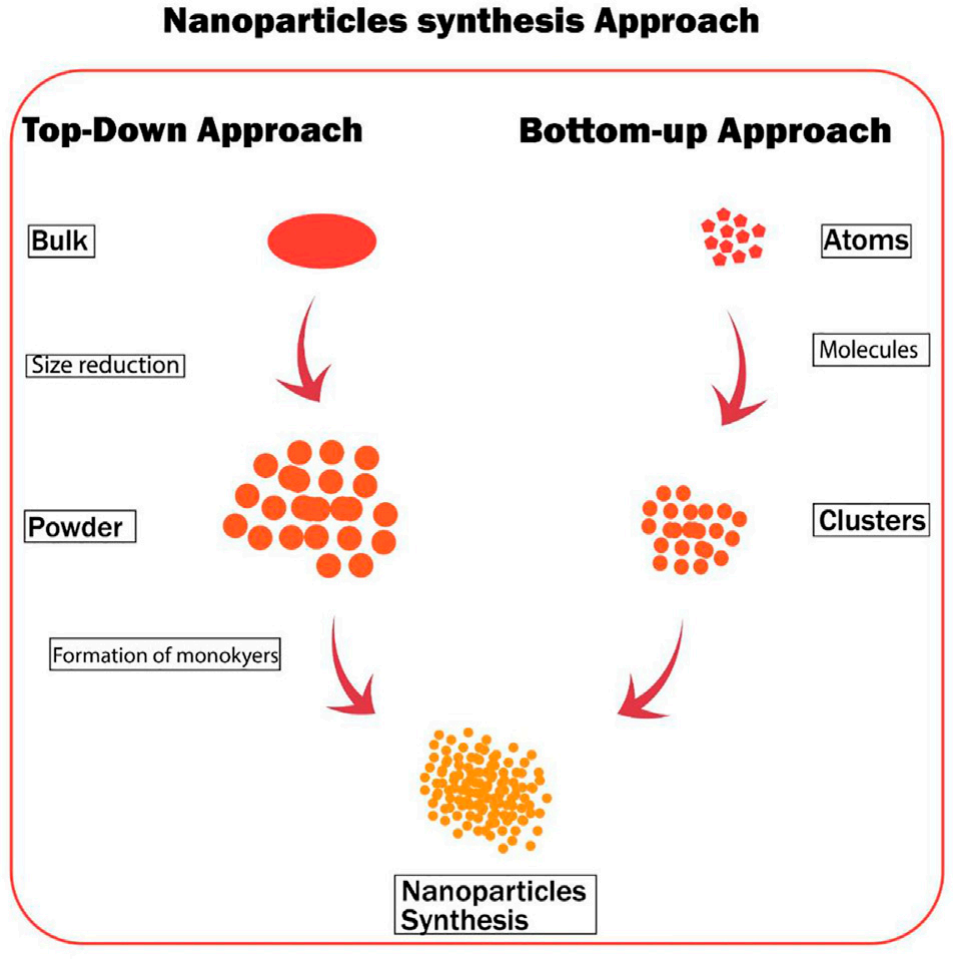
Physical and chemical approaches of nanoparticles synthesis.

In this approach, bulky materials are divided into smaller materials to produce NPs (Figure 2). In etching or lithography, the electrons or beam of light is used for developing nanoarchitectures, which are further divided into masked and maskless lithography. In masked lithography, templates or masks are used to transfer the nanopatterns, which further includes soft lithography (Yin et al., 2000), photolithography (Szabó et al., 2013), and nanoimprint lithography (Kuo et al., 2003). In maskless lithography, nanopattern writing is performed without any mask, which further consists of electron beam lithography, ion beam lithography, and scanning probe lithography. Ion implantation can be used to perform micro-nano-fabrication with a focused ion beam along with chemical etching. Electro-explosion is the simplest method for the synthesis of nanostructured materials, which is being commonly used for the synthesis of nanofibers like polymers. The advanced method of electrospinning is coaxial electrospinning, in which two capillaries are used as a spinneret. Two viscous liquids or non-viscous liquids are used as core and viscous as shell to synthesize core–shell nanoarchitectures in an electric field. This technique is used to synthesize ultrathin fibers (Baig et al., 2021).

**FIGURE 3:**
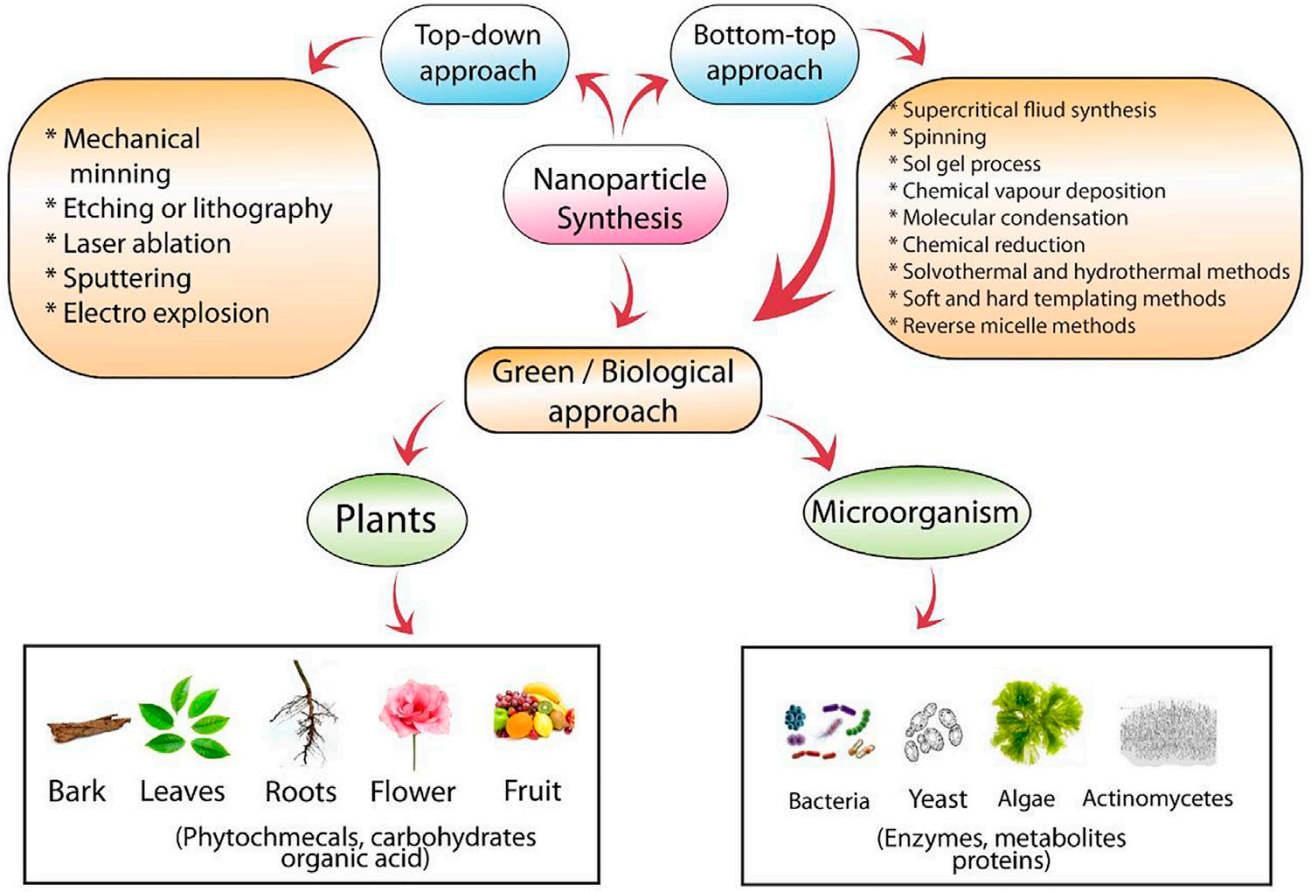
Physical, chemical, and biological approaches for the synthesis of nanoparticles.

Mechanical milling is used to produce nanocomposites (Figure 3). Nanocomposites produced from this method are Ni, Cu, Al, or Mg-based nanoalloys, carbide-strengthened aluminum alloys, oxide-strengthened aluminum alloys, wear-resistant spray coating, and many other nanocomposites (Yadav et al., 2012). In sputtering, high-energy gas or plasma particles are bombarded onto a solid surface to produce nanomaterials. The method is used to produce thin nanofilms. Various ways are available for the sputtering process to carry on like DC diode sputtering, magnetron, and radio-frequency diode. Magnetron sputtering is used to produce carbon paper substrates and WSe2-layered. The NPs made using this technique had the same composition as the targeted material with little impurities, and it is also cost-effective (Nie et al., 2009; Nam et al., 2020). In laser ablation, NPs are manufactured by the bombardment of a laser beam at the targeted material to produce vapors. This technique is also known as green synthesis as it does not require any stabilizing agent (Figure 3). Nanomaterials produced by this technique are carbon nanomaterials, ceramics, oxide composites, and metal nanoparticles (Baig et al., 2021).

### Bottom-Up Approaches

A bottom-up approach is the accumulation of a substance from the bottom, that is, atom-by-atom, molecule-by-molecule, or cluster-by-cluster (Benelmekki, 2015). Chemical vapor deposition (CVD) is used to produce low cost, good stability, free of impurities, long shelf-life, and non-hazardous carbon nanotubes. In this method, a gas containing carbon is used as a precursor, which upon decomposition produces carbon atoms (Machac et al., 2020). Similarly, the hydrothermal method is performed in heterogeneous reactions in an aqueous medium, while the solvothermal method is performed in a non-aqueous medium at high temperature and pressure in a sealed vessel (Chen and Holt-Hindle, 2010). These methods are used to produce nanorods, nanospheres, nanowires, and nanosheets (Chai et al., 2018; Dong et al., 2020).

**FIGURE 4:**
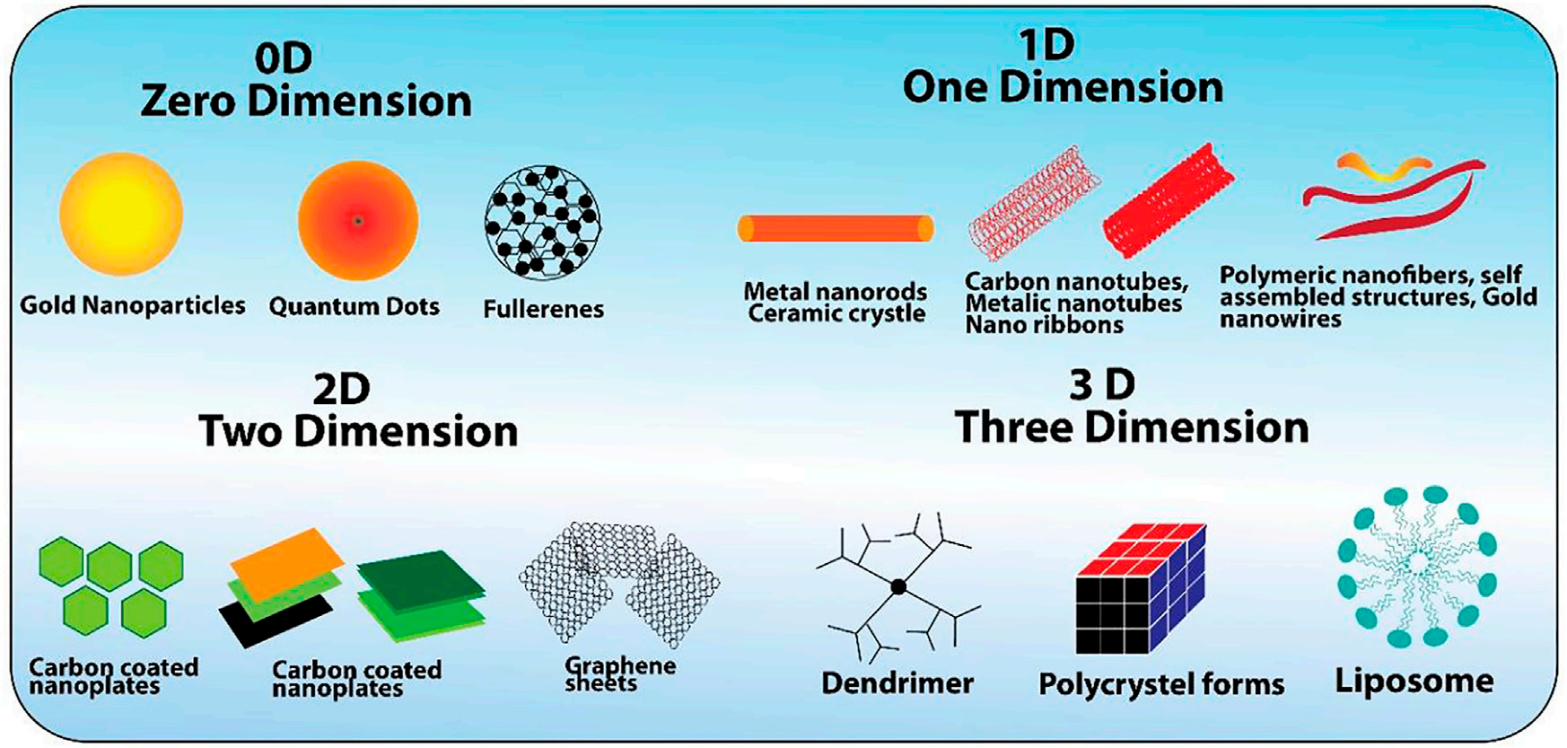
Classification of nanomaterials on the base of size and shape.

Soft and hard templating methods are used to produce nanoporous materials. In the soft templating method, the NPs are produced using many soft templates like flexible organic molecules, cationic, non-ionic, and anionic surfactants, and block copolymers. The soft templating technique is used to produce 3D ordered mesoporous materials such as cubic (MCM-48), hexagonal (MCM-41), ordered mesoporous silicas, and lamellar (MCM-50) (Figure 4). Hard template/nano-casting is used to synthesize silica, colloidal crystals, carbon black, particles, wood shells, and carbon nanotubes (Baig et al., 2021). In reverse micelles, water-in-oil emulsions are used for the synthesis of NPs. The size of the nanoparticles is based on the size of these nanoreactors and the concentration of the water. If water is in large amount, the size will be greater, and *vice versa* (Malik et al., 2012; Nguyen, 2013). In a sol-gel method, the precursor used is a sol such as metal alkoxide, which is readily transformed into a network structure called gel (Baig et al., 2021). This method is widely used to produce composites, powder, and film nanostructures (Parashar et al., 2020).

## Characterization of Nanomaterials

**FIGURE 5:**
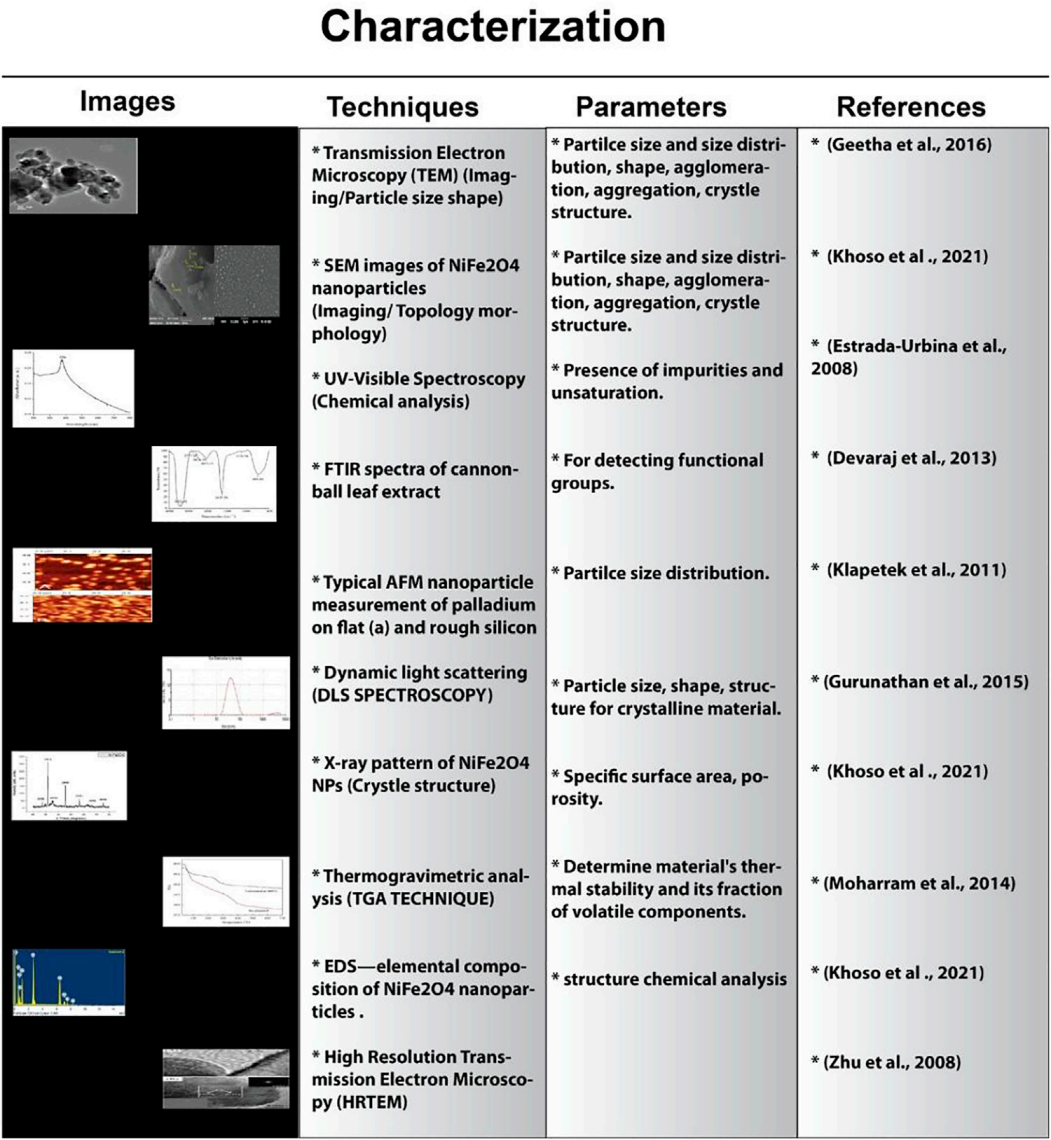
Methodologies involved in the characterization of nanoparticles.

Several techniques are used to characterize the NMs. Atomic-scale images of the crystallographic structure of the nanoparticles are obtained with the help of a transmission electron microscope (TEM) (Frenkel et al., 2001). Similarly, high-resolution transmission electron microscopy (HRTEM) is employed for the determination of arrangements of atoms and their local structures, such as lattice vacancies, screw axes, lattice fringe, defects, glide plane, and the arrangement of the surface atomic structures (Benelmekki, 2015). A 2D image with spatial variations can be obtained with the help of scanning electron microscopy (SEM), in which the data for the selected areas of the surface of the NMs can be collected (Zafar et al., 2021). Atomic force microscopy (AFM) is used to capture 3D pictures for quantitative measurement of NMs both in liquid and gas phases to measure volume, width, height, and length, along with the surface texture and morphology at the submicron level with inbuilt image processor software (Figure 5) (Vahabi et al., 2013).

The nanoparticles ranging from 2 to 100 nm are characterized by using ultraviolet–visible spectroscopy (UV-VIS) (Zafar et al., 2018). Dynamic light scattering (DLS) spectroscopy is used for surface charge measurement and the size of the Brownian NPs in a colloidal solution (Tomaszewska et al., 2013). The bonding characteristics of the various nanoparticles, the structure of the elements present in the magnetic nanoparticles, and the mechanism of the reactions occurring at the surface of the nanoparticles can be determined by X-ray diffraction (XRD) (Benelmekki, 2015). Thermogravimetric analysis (TGA) is used to explore the binding efficiency and composition of the coatings of the nanoparticles such as polymers or surfactants. Raman spectroscopy (RS) is used to determine the vibrational signals of the chemical species which are adsorbed at the surface of the NPs while synthesizing. In addition, it can be used to identify the molecular attachment of a single molecule on the surface of silver NPs (Ali et al., 2021). Fourier transform infrared spectroscopy (FTIR) is used to identify the functional groups such as hydroxyl (OH^−^) and carbonyl (C=O) moieties that are adsorbed on the surface of the products in nanoparticle synthesis (Figure 5) (Mourdikoudis et al., 2018).

## Applications of Nanotechnology in Agriculture

### Nano-Sensors

Nanosensors act as nano-carriers envisaged to replace the agrochemicals due to their biological compatibility, less soil contamination, efficiency, and precise release of active substances, increased solubilization, and penetration into plant and target tissues (Prasanna et al., 2021). Nanosensors are chosen over the conventional sensors due to nanoscale size, highest sensitivity, efficient signal transduction mechanism, turn off/on mechanism, and durability (Scognamiglio, 2013). On insect bite, biosynthesis of detectable volatile compounds is stimulated in host plants which are detected by wireless nanosensors (Afsharinejad et al., 2015). Floating bio-nanosensors in photosystem II of the chloroplast detect organo-phosphates, neonicotinoids, carbamates, atrazine pesticides, and transmit signals (Přibyl et al., 2006). Similarly, normal glutathione-regulated AuNP-based bio-nanosensor and ceramic-coated nano-biosensors integrated with Ag-Pd electrodes are being widely employed in the detection of Cd^2+^ toxicity (Fang et al., 2010).

Electrochemical and electromagnetic signals are precisely detected by electrical nanosensors. These kinds of nanosensors are used to detect the pesticide residues in food and environment, quality assurance, remote sensing of cultivated crops, and climatic conditions. For example, Ag/Au-plated carbon electrodes integrated nanosensors are being used in the detection of triazophos, methyl parathion, and organophosphorus insecticidal residues in the post-harvest vegetables (Zhang et al., 2013). Optical sensors are being employed in the detection of heavy metals in freshwater bodies and soil. Nanosensor-based global positioning system (GPS) has enabled remote monitoring of plant growth and auto-irrigation. 1D nanofibers such as potassium niobate (KNbO3) are being used to measure the level of humidity (Ganeshkumar et al., 2016). The major constraint in the acceptability of nanosensors is their cost due to high-tech machinery and inputs.

### Nano-Fertilizers

**FIGURE 6:**
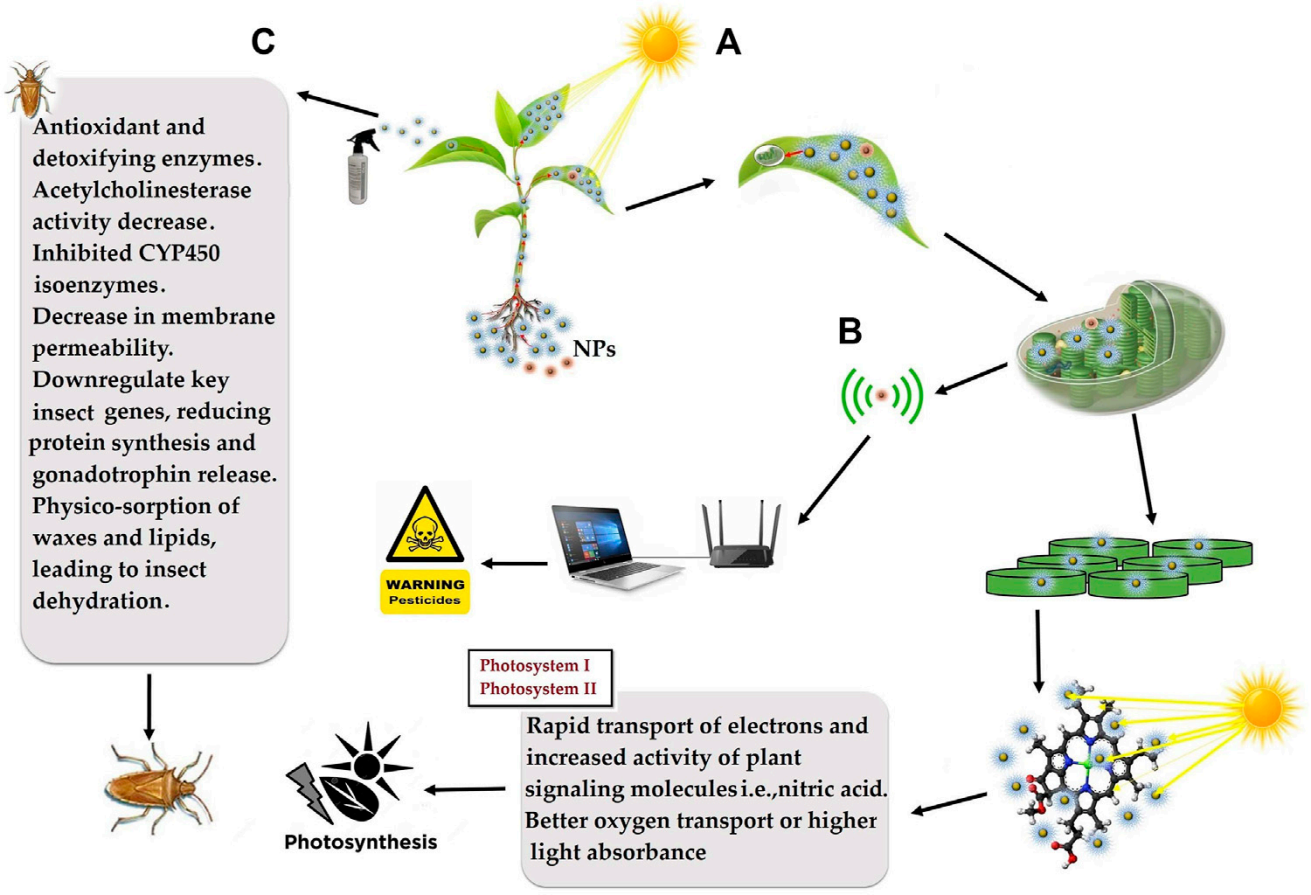
Schematic diagram of nanoparticles application for; **(A)** enhanced light harvesting to accelerate the rate of photosynthesis for increased biomass accumulation and crop yield, **(B)** nanobionics application in early detection of pathogenic infection and pesticidal poisonous residues for improved biosafety, and **(C)** slow-release nanopesticides application for precise and durable pest management.

Crop yield can be increased by 35–40% through adequate fertilizer management, irrigation, and use of quality seeds (Shekhawat et al., 2012). Overdosing of conventional fertilizers is contaminating soil, freshwater bodies, and their nutrient use efficiencies are also very low, that is, N ∼35%, P ∼20%, and K ∼40% (Rosell and Sanz, 2012). Nanofertilizers remain fixed in soil for a long time for the complete absorption of nutrients by plants. Nanofertilizers are slow release, control loss, and magnetic in nature which may contain Zn, SiO_2_, Fe, TiO_2_, ZnS, and Mn/ZnSe. Metal oxides such as CeO_2_, ZnO, TiO_2_, Al_2_O_3_, FeO, and ZnO are also being used as nanofertilizers (Sabir et al., 2014). Nano-ZnO- and nano-sulfur-coated monoammonium phosphate and urea displayed slow release of Zn and S to enhance their uptake efficiency (Wilson et al., 2008; Milani et al., 2012). Similarly, nitrogen release was twice that of conventional urea fertilizer when hydroxyapatite-NPs were used to coat urea and encapsulate it into softwood cavities (Kottegoda et al., 2011). Nanofertilizers are easily absorbed by roots and leaves and have numerous advantages over the traditional fertilizers (Figure 6).

Nanofertilizers are designed by encapsulation of cationic nutrients such as Ca^2+^, K^+^, NH^4+^, and Mg^2+^ or anionic nutrients such as SO^4−^, PO^4−^, and NO^3−^ in nanoparticles, nanoporous materials, or coated on thin films. Nanofertilizers such as NH_3_-zeolites and K-graphene oxide enhanced P and K use efficiency by avoiding leaching and the precise release of nutrients in the required amount. Similarly, the application of nano-calcite (CaCO_3_) along with Fe_2_O_3_, SiO_2_ and MgO nanoparticles enhanced the uptake of Ca, Fe, Mg, P, Zn, and Mn (Sabir et al., 2014). The application of ZnO nanofertilizer to barley crop resulted in enhanced photosynthesis rate, root length, shoot length, and 60% higher yield as compared to the traditional ZnSO_4_ fertilizer (Kale and Kane, 2017). The application of carbon dots together with traditional fertilizers resulted in an increased yield of soybean by 16.74%, rice by 10.29%, maize by 10.93%, and wheat by 28.81% (Iqbal and Umar, 2019). Long time or overuse of nanofertilizers can contaminate the soil and may have some serious health issues in animals and human beings.

### Nano-Pesticides

Conventional pesticides are being widely sprayed to control insect pests but only 0.1% of active ingredients reach the target pest (Carriger et al., 2006). Biopesticides are cheap and eco-friendly alternatives of synthetic pesticides, but their mode of action is comparatively slow. Nanopesticides are robust due to the slow and controlled release of poisons to provide durable insect control (Kah et al., 2019). Nanopesticides in low concentration are more effective such as 9.86 mg/L of 50% SDS/Ag/TiO_2_-IMI can control 100% *Martianus dermestoides* as compared to 13.45 mg/L of 95% conventional IMI (Guan et al., 2008). Nano-permethrin, nano-acephate, and nano-carbofuran efficiently reach target pests as compared to conventional permethrin, acephate, and carbofuran (Pradhan et al., 2013). SiO_2_–chlorfenapyr controlled cotton bollworms two times higher than chlorfenapyr microparticles (Wu et al., 2017). Nowadays, nanopesticides are being commercially produced by some multinational companies such as Primo Maxx®, Subdue Maxx®, Banner Maxx® II, Karate Zeon, Penncap-M®, Syngenta, and BASF (Gouin et al., 2008). The application of Ag-SiO_2_ myco-nanoparticles controlled the following pathogenic fungi *Rhizoctonia solani*, *Colletotrichum gloeosporioides*, and *Magnaporthe grisea*, and Raffaelea species in oak trees (Alghuthaymi et al., 2015). A mixture of trifloxystrobin 25% and tebuconazole 50% nano-fungicides controlled the soil-borne fungus *Macrophomina phaseolina* (Singh et al., 2017). Nanopesticides are costly and not in reach of common farmers.

### Nano-Herbicides

The conventional herbicides are expensive, contaminate the environment, and are hazardous to health, and seize crop growth. Nano-herbicides are a good choice to mitigate the disadvantages of conventional herbicides by precise and targeted delivery and easy to degrade. Nano-atrazine, nano-ametryn, nano-simazine, and nano-paraquat were synthesized *via* encapsulation in poly (epsilon caprolactone) to enhance their activity, durability, avoid leaching, and toxicity (Anderson et al., 2018). Effectiveness of nano-atrazine against *Amaranthus viridis* and *Bidens pilosa* is many folds high as compared to conventional atrazine (De Souza et al., 2021). Nano-emulsion of glyphosate revealed its enhanced efficacy as compared to the commercial formulation (Lim et al., 2013). Conventional herbicides also have serious consequences over beneficial insects such as earthworms exposed to conventional atrazine displayed vacuolization, pyknotic cells, and dismantled epithelial tissues, and chloragogenous layer (Oluah et al., 2010). On the other hand, nano-herbicides resulted in lower toxicity to *Pseudokirchneriella subcapitata* and *Prochilodus lineatus* (Andrade et al., 2019).

### Nano-Fungicide

Pathogenic fungi such as *Alternaria alternata*, *Fusarium oxysporum*, *Phoma destructiva*, and *Cochliobolus lunata* cause diseases in plants and resulted in severe yield loss. NMs are being employed in unraveling plant–microbe interaction networks. Nano-fungicides precisely and efficiently mitigate pathogenicity caused by fungal pathogens as compared to conventional fungicides. For example, application of nano-Ag causes severe halt in hyphae, conidiophores, and conidia development of powdery mildew which causes serious damages to cucurbits family (Lamsal et al., 2011). Similarly, the application of TiO_2_ and ZnO NPs seize the growth of a broad range of plant fungal pathogens such as *Penicillium expansum*, *Botrytis cinerea*, and fungal pathogens of litchi (Lu et al., 2006). The biggest challenge in use of nano-fungicides is the unavailability of established dosimetry and high cost.

### Nanomaterials Application at Post-harvest Level

Green-synthesized Au/Ag-NPs along with edible coating are being employed in maintaining the long-lasting shelf life of fruits and vegetables to refrain from potential post-harvest loss causing phytopathogens (Nxumalo and Olaniyi, 2022). Post-harvest losses are predominantly caused by the following two insect groups: Coleoptera and Lepidoptera. The application of nano-Ca on fruits and vegetables has proved very effective to control oriental fruit fly and red scale insects (Kitherian, 2017). Similarly, CaCO_3_-NPs were used as fertilizers to improve the nutritional value of plants and insecticides. Spraying of SiO3-NPs effectively controlled larvae of Asian armyworm (Kitherian, 2017). The modification of Ag-electrodes with Cu-NPs in nanosensors help in early detection of karnal bunt disease in wheat and real-time measurement of salicylic acid (SA) contents in oil extracted from seeds (Kashyap et al., 2017). Spreading of a handful of nano-Al_2_O_3_ on wheat grains thoroughly killed the following two insect pests: *Stiophilus oryzae* and *Rhyzopertha dominica* (Stadler et al., 2010). To avoid harmful effects of conventional poisonous pesticides over human health, essential oil extract from anise (*Pimpinella anisum*) in the form of nano-emulsion is being used to control flour beetle (*Tribolium castaneum*) and nano-SiO_2_ to control insect pests during grain storage (Gamal, 2018; Rangasamy et al., 2018). Although, the NP application in the field of post-harvest loss has gained notable attention, the major limitations in their commercialization are production cost and potential health risks.

### Nanomaterials for Farmland Restoration

The cultivable and fertile farmlands are going to be barren due to over-cultivation, water scarcity, and global warming. NMs have potential in the restoration of farmlands such as hydrogel (potassium polyacrylate), nano-clays, and nano-zeolites can enhance the water holding capacity of soil by 50–70% and reduce soil compactness by 8–10% (Sekhon, 2014). Water-insoluble polymers such as hydrogel application in rainfed land enhanced water use efficiency by avoiding leaching, improved soil texture, evaporation, microbial activity, and resulted in increased crop yield. NMs are eco-friendly, non-toxic to plants, and rapidly degraded into nitrogen, water, and CO_2_. Similarly, heat-repellant fertilizers such as nano-zeolites increase water holding capacity, nutrient use efficiency, soil aeration, microbial growth, and soil decontamination by absorbing heavy metals (Saponaro et al., 2016).

### Nanomaterials as Drought-Tolerant Agents

Drought stress can lead to the production of oxygen radicals that result in increased lipid peroxidation and oxidative stress in the plants. Visible effects include stunted growth, narrow leaves, damaged foliar matrix, and decreased biomass contents. Hydrogel can reduce the drought impact on plants leading to reduced stress and oxygen radical formation. This in turn provides scope for better growth and yield even in unfavorable climatic conditions.

### Nanomaterials as Growth Enhancers

Irrigation technology has major constraints in the fields of application of fertilizers, herbicides, and germicides. Studies suggest that the use of synthetic fertilizers can be greatly reduced when hydrogel agriculture is practiced without hindering crop yield and nutritional value. It would indeed be more appropriate practice for sustainable agriculture in arid and semi-arid conditions and regions with similar ecological constraints. Moreover, potassium polyacrylate is safe and non-toxic, thus preventing pollution of agro-ecosystems.

### Nanomaterials Induced Biomass Accumulation

Carbon dots have a size range between 1–10 nm along with having fluorescent properties. Carbon dots absorb a range of UV light, get excited, and express usually bluish in color at neutral pH. By taking into account the biomass production of any crop or plant can be increased through the application of carbon dots which express blue color. Applied carbon dots are first absorbed by plant roots, later transported to aerial parts and enter cellular organelles, especially chloroplast. In the thylakoid membrane, the carbon dots absorb blue light similar to other pigments present in the photosystem I or II and transfer the obtained energy to the main reaction. The sunlight absorbed by these carbon dots enhances photosynthesis, which ultimately leads to high biomass production (Figure 6). For example, the application of single-walled carbon dots enhance the rate of near-infrared fluorescence light-harvesting, rate of electron transfer by 49%, and downregulate ROS in chloroplast resulting in increased photosynthetic efficiency and crop yield (Giraldo et al., 2014). The application of nano-TiO_2_ resulted in an increased rate of photosynthesis of spinach by 3.13 times (Zheng et al., 2005). At present, the development of nano-robots and their delivery is challenging which further needs improvement.

### Nanoparticles Mediated Cell Culture and Gene Delivery

In order to get the desired aesthetic traits, biotic, and abiotic stress resistance, higher crop yield, and increased biomass for the paper and pulp industry, genetic transformation is inevitable. The exposure of plant cells to NPs perturbs genetic expression, alters biological pathways, and affect plant metabolism (Nair, 2012). The delivery of RNAi constructs coated NPs, including chitosan, cationic dendrimers, and liposomes in insects to target lethal genes for insect control is a promising tool in sustainable crop yield (Yan et al., 2021). SiO_2_-NPs are being widely employed in gene transformation in animal cells, but it is not successful in plants cells due to the hard cell wall. Recently, gold encapsulated mesoporous SiO_2_-NPs have been developed which can pass through the cell wall of plants (Torney et al., 2007). Similarly, a cell culture of tobacco can be increased 55–64% by supplementing with multi-walled carbon nanotubes (Khodakovskaya et al., 2012).

## Agro-Ecological Risks

### Soil Contamination

Soil, a primary recipient of nanofertilizers, is subjected to contamination and side effects caused by chemical processes by NPs and have serious consequences over soil microflora, microfauna, injuries to earthworms digestive tract, and consequently soil fertility (Dubey and Mailapalli, 2016). The properties of nanoparticles may change the structure of the soil and default to detect contamination due to nanoparticles in soil and environment (Lacave et al., 2020). The size of NPs determines their ecotoxic effects such as TiO_2_ with 7-nm surface area and CeO_2_ with 15-nm surface area are highly toxic to nematode (*Caenorhabditis elegans*) as compared to comparatively larger size TiO_2_ with 20 nm and CeO_2_ with 45-nm surface area, respectively (Roh et al., 2010). If doses are increased from a certain amount, ZnO nanoparticle becomes toxic to the soil. Similarly, the application of a higher concentration of ZnO such as 5 g/kg of soil resulted in over-accumulation of ZnO in the body of earthworm (*Eisenia fetida*) and resulted in DNA damage (Hu et al., 2010).

### Phyto-Toxicity

In plants, toxic nanoparticles cause severe halt in respiration and photosynthesis (Navarro et al., 2008). The toxicity caused by TiO_2_ nanoparticles is more severe on algae than daphnids (Hund-Rinke and Simon, 2006). Nanoparticles of TiO_2_ retain in the soil for a long time, stick with the cell wall of the wheat plant, and resulted in halt in biomass (Du et al., 2019). The corn, lettuce, and rye grass seedlings exposed to Al_2_O_3_ nanoparticles displayed stunted roots, while radish and rapeseed displayed elongated roots (Lin and Xing, 2007). Rice seedlings exposed to AgNPs displayed damaged cell walls due to the transport of nanoparticles (Mazumdar and Ahmed, 2011). Notably, ZnO-based nanoparticles remain fixed in the soil for a longer time and are toxic to plants. For example, rice seedlings exposed to ZnO nanoparticles displayed stunted root growth and severe halt in biomass contents (Boonyanitipong et al., 2011). Uncoated Al_2_O_3_-NPs application on corn, soybean, cucumber, carrot, and cabbage resulted in stunted roots (Yang and Watts, 2005).

### Water Contamination

The leaching of toxic nanoparticles such as Ag, Cu, Al, Ni, TiO_2_, and Co in freshwater bodies and air is very toxic to humans, animals, and aquatic life. Notably, toxicity caused by nanoparticles in freshwater bodies and air is irreversible. Metal-based nanoparticles are less toxic as compared to their oxides such as TiO_2_ which causes severe halt in the rate of photosynthesis in algal cells (Sharma et al., 2009). Nanoparticles made of oxides of Ag, Fe, and Cu enhance the rate of mortality, premature hatching, and heart diseases in zebra fish (Zhu et al., 2012).

### Human Health Risks

The exposure to nanoparticles through inhalation of air, ingestion through the gastrointestinal tract, and dermal contact may cause serious health risks. Poor ventilation system during manufacturing is a serious cause of inhalation of nanoparticles (Gaffet, 2006). Inhaled nanoparticles of size ≥50 nm pass through the gastrointestinal tract and are transported to the spleen, liver, blood, and finally the bone marrow (Hagens et al., 2007). Originally, the skin is a barrier against pests and contaminants but the injured skin due to dermatitis, eczema, and irritation let nanoparticles penetrate the body which may cause edema, erythema and, eschar formation.

## Dosimetry of Nanomaterials to Ensure Biosafety

Indeed, it is mandatory to determine the optimum level of concentration of any new material which must be safer to use. Therefore, the required dose of NMs was determined by lethal dose 50 (LD_50_). Through LD_50_, the level of cellular toxicity is determined that at what concentration of NPs cause 50% cell mortality and 50% cell viability. The poisoning potential of NPs can be measured through a variety of assays such as 3-(4,5-dimethylthiazol-2-yl)-2,5-diphenyltetrazolium bromide (MTT) (Raja et al., 2019), terminal deoxynucleotidyl transferase dUTP nick end labeling (TUNEL) apoptosis assay (Jin et al., 2021), Alamar blue assay (Usman et al., 2020), sulforhodamine B (SRB) (Massard et al., 2018), and cell counting kit-8 (CCK-8; Dojindo Molecular Technologies) (Jin et al., 2021) can be used to determine the cellular toxicity (mortality or viability percentage). *In vivo* application of NPs in agriculture can cause genotoxicity to both terrestrial and aquatic animals which can be measured using sodium dodecyl polyacrylamide gel electrophoresis (SDS-PAGE) assay (El-Sappah et al., 2022). Immunotoxicity caused by NPs can be determined by pro-inflammatory and anti-inflammatory cytokines (Engin and Hayes, 2018). NPs can be employed in the electrochemical detection of heavy metals in agriculture and seafood to ensure biosafety (Aragay and Merkoçi, 2012).

## Conclusion and Future Perspectives

Agri-nanotechnology has revolutionized the designing and application of nanoparticles of size 1–100 nm in agriculture and forestry. Nanofertilizers such as SiO_2_-N remain fixed in the soil for a long time to enhance fertilizer use efficiency and avoid soil and water contamination to ensure biosafety. Nanobionics has enabled the synthesis of nano-robots which can pass through the cell membrane, translocate among organelles, capture photos, and transmit *via* wireless devices. Nanosensors are being employed in the detection of pesticide residues in fruits, vegetables, and seed oil at the post-harvest level to avoid health risks. A thin lining of green-synthesized edible NPs inside metal packaging improves shelf life, maintains original taste and nutrition, avoids corrosion of metal particles, and ensures health benefits. Nanosensors are being employed in unraveling plant microbe interaction and mitigation of pathogenicity to ensure the biosafety. Hydrogel polymers and nano-zeolites have water holding, heat resistance, enhanced soil aeration, and microbial growth capacity to tolerate drought and heat stress as well as farmland restoration. The prime cause of failure of genetic transformation is landing of construct in junk DNA, which is 70% of the total. Gold capped nano-SiO_2_ particles have potential in chromosome and site-specific gene delivery. GPS-based nano-devices have enabled remote sensing of agro-forestry cultivation, auto-irrigation, and safety. Among forestry products, cellulose is of high importance because of its use as feedstock in the paper and pulp industry. In photosystems I and II, bionic nanosensors potentially enhance light harvesting and biomass accumulation. Today, the commercial application of NPs in agriculture and forestry is not common due to expensive raw materials and the requirement of high-tech machinery. With the improvement in technology, NP-based agrochemicals will be readily available to farmers, which will contribute much to the improvement of yield, nutritional value, shelf life, and biosafety.

## Data Availability

The datasets presented in this study can be found in online repositories. The names of the repository/repositories and accession number(s) can be found below: GSE192458.
